# Effect of Introducing a Mini-Ergometer to the Ward Due to the COVID-19 Pandemic-Imposed Restrictions on Rehabilitation Centers on Physical Function: Cardiac Rehabilitation of Patients with Heart Failure

**DOI:** 10.3390/jcm14175922

**Published:** 2025-08-22

**Authors:** Etsuko Mori, Hideki Ishii, Hirotaka Matsuura, Yuji Kono, Yoichiro Aoyagi, Rio Shimizu, Tomoyasu Hiratsuka, Yoshihiro Sobue, Eiichi Watanabe, Hitoshi Kagaya

**Affiliations:** 1 Department of Rehabilitation, Fujita Health University Bantane Hospital, Nagoya 454-8509, Aichi, Japan; rio970714@gmail.com (R.S.); hira@fujita-hu.ac.jp (T.H.); 2 Department of Cardiovascular Medicine, Graduate School of Medicine, Gunma University, Maebashi 371-8511, Gunma, Japan; hkishii@gunma-u.ac.jp; 3 Department of Rehabilitation Medicine, School of Medicine, Fujita Health University, Toyoake 470-1192, Aichi, Japan; hero850411@yahoo.co.jp (H.M.); yyy@rc5.so-net.ne.jp (Y.A.); hkagaya2@ncgg.go.jp (H.K.); 4 Department of Rehabilitation, Fujita Health University Hospital, Toyoake 470-1192, Aichi, Japan; yt-kono@fujita-hu.ac.jp; 5 Department of Rehabilitation Medicine, Graduate School of Medicine, Nippon Medical School, Tokyo 113-8602, Japan; 6 Division of Cardiology, Department of Internal Medicine, Fujita Health University Bantane Hospital, Nagoya 454-8509, Aichi, Japan; sobue@fujita-hu.ac.jp (Y.S.); enwatan@fujita-hu.ac.jp (E.W.); 7 Department of Rehabilitation Medicine, National Center for Geriatrics and Gerontology, Obu 474-8511, Aichi, Japan

**Keywords:** cardiac rehabilitation, inpatient rehabilitation, physical performance, COVID-19, controlled training, ward rehabilitation, ergometer exercise, heart failure, rehabilitation unit

## Abstract

**Background/Objectives**: The COVID-19 pandemic restrictions had negative effects on cardiac rehabilitation. The difference in the location of cardiac rehabilitation practice during the COVID-19 pandemic decreased the amount of aerobic exercise and the patient’s physical function at discharge. Therefore, we introduced a mini-ergometer to the ward to provide quantitative aerobic exercise. This study aimed to evaluate physical function at discharge after the introduction of a mini-ergometer to the ward in patients with heart failure. **Methods**: We included a total of 117 consecutive patients who were admitted to a university hospital and underwent a cardiac rehabilitation program for heart failure between June 2020 and September 2022. Patients were divided into two groups: Group A (*n* = 54), which included those admitted before the introduction of the mini-ergometer, and Group B (*n* = 63), which included those admitted after the introduction of the mini-ergometer. Data, including age, sex, and functional status, were obtained. Additionally, the duration of each rehabilitation exercise was measured. **Results**: Group B had a significantly higher 6 min walking distance at discharge than Group A (Group A: 236.0 m vs. Group B: 290.0 m, *p* = 0.020). Furthermore, Group B had a significantly longer ergometer exercise duration than Group A (Group A: 0 min vs. Group B: 25.0 min, *p* < 0.001). **Conclusions**: The results showed that the introduction of the mini-ergometer to the ward could ensure quantitative exercise loads even under restricted access to rehabilitation centers during the COVID-19 outbreak, thereby improving physical function at discharge in patients with heart failure.

## 1. Introduction

Aerobic exercise during cardiac rehabilitation is strongly recommended for patients with cardiovascular diseases [1]. It is a cornerstone of exercise prescription and plays a crucial role in rehabilitation. Beneficial effects of aerobic exercise in patients with cardiovascular disease have been demonstrated, including (1) improvement in left ventricular ejection fraction (LVEF) [2], (2) reduction in hospital readmission rates [3], (3) enhancement of quality of life [3], (4) the advantage of improving exercise tolerance [3,4,5], (5) improving walking speed [6], and (6) enhancing muscle strength [7]. Aerobic exercise in cardiac rehabilitation programs is mainly performed using a bicycle ergometer or treadmill installed in rehabilitation centers [8].

The COVID-19 pandemic-imposed restrictions on cardiac rehabilitation [9,10,11]. Kida et al. [11] conducted a survey involving 37 cardiac rehabilitation facilities in Japan during the early stage of the COVID-19 pandemic and reported that 49% of the facilities had temporarily suspended group exercise rehabilitation. However, all facilities maintained individual inpatient cardiac rehabilitation [11]. The Japanese Association of Cardiac Rehabilitation recommends cardiac rehabilitation standard programs for conducting exercise therapy in the rehabilitation unit after the acute phase mobilization program [12]. However, cardiac rehabilitation programs were implemented mainly in wards instead of rehabilitation centers to avoid the “three Cs,” including crowded places, close-contact settings, and confined and enclosed spaces [13]. In our previous study [14], the effect of modified rehabilitation therapy performed in wards was compared with that of conventional therapies performed in rehabilitation centers where exercise on a treadmill and cardiopulmonary exercise testing were available. Patients admitted during the COVID-19 outbreak showed a significantly shorter ergometer exercise time, lower 6 min walking distance (6MWD), and lower walking speed at discharge than those admitted before the COVID-19 outbreak [14]. The difference in the location of cardiac rehabilitation practice during the COVID-19 pandemic might have affected the rehabilitation modality and the patient’s physical function at discharge. Therefore, a mini-ergometer was introduced to the ward to provide quantitative aerobic exercise, even under restricted access to rehabilitation centers. We hypothesized that the introduction of a mini-ergometer to the ward would lead to greater improvement in physical function at discharge. Therefore, this study aimed to compare physical function at discharge before and after the introduction of a mini-ergometer to the ward in patients with heart failure.

## 2. Materials and Methods

### 2.1. Patients

Emergency patients who were admitted to our hospitals between June 2020 and September 2022 due to worsening heart failure and underwent cardiac rehabilitation were enrolled in this study. The inclusion criteria were patients aged 18 years and those who could complete the 6MWD measurement at discharge. The exclusion criteria included those with a history of COVID-19 infection, those who were readmitted during the study period, those with severe dementia, those with a history of neuromuscular disease, and those who did not wish to participate. In order to exclude acute infection, COVID-19 testing was performed for all patients at the time of admission.

The patients were divided into two groups: those admitted before the introduction of the mini-ergometer (Group A, April 2020–June 2021) and those admitted after the introduction of the mini-ergometer (Group B, July 2021–September 2022). Based on previous reports, the expected difference in 6MWD between groups was approximately 80 m, and the estimated standard deviation was around 190 m, resulting in a Cohen’s d of approximately 0.42 [14]. Under these conditions (α = 0.05, power = 0.80), the required sample size is estimated to be between 110 and 120 participants. Therefore, the sample size was regarded as appropriate for the purposes of this study.

### 2.2. Study Design and Protocols

This was a retrospective observational study conducted using our single-center database. This study was conducted in accordance with the principles of the Declaration of Helsinki and approved by the Research Ethics Committee of the institution (Approval No.: HM20-538). Informed consent was obtained using the opt-out method approved by the Committee.

### 2.3. Data Collection

Patients’ demographic and clinical characteristics were obtained from the medical records. During hospitalization, the patients’ demographic and clinical characteristics were evaluated, including blood investigation, heart failure severity, cardiac function, physical function, cognitive function, activities of daily living (ADL), and cardiac rehabilitation program. Additionally, age, sex, systolic blood pressure at admission, body mass index, comorbidities, and medication were recorded as demographic characteristics. Each patient’s serum hemoglobin, albumin, and sodium concentrations were obtained, and the glomerular filtration rate was estimated from the blood investigation results at admission. The geriatric nutritional risk index was calculated from individually obtained serum albumin levels and body weights as follows [15]: geriatric nutritional risk index = [14.89 × albumin (g/dL)] + [41.7 × (body weight/ideal body weight)]. These blood parameters were obtained from samples collected in the outpatient laboratory at the time of hospital admission.

### 2.4. Heart Failure Severity and Cardiac Function

Evaluation of HF severity was conducted using N-terminal pro-brain natriuretic peptide (NT-proBNP). Cardiac function was evaluated by LVEF, a parameter of the left ventricular systolic function obtained via echocardiography and New York Heart Association (NYHA) functional class. Echocardiographic assessments were performed by one medical technologist per patient. Upon completion of the assessment, the findings were reviewed and approved by a cardiologist.

### 2.5. Physical Function

Physical function was assessed 3 days before discharge using the 6MWD, grip strength, isometric knee extensor muscle strength (KEMS), walking speed, functional reach test, and waist circumference. Additionally, grip strength and walking speed were assessed at the start of cardiac rehabilitation. The 6MWD test was performed in accordance with the guidelines of the American Thoracic Society [16]. Grip strength was measured using a digital grip strength dynamometer (T.K.K.5401, Takei Scientific Instruments, Niigata, Japan). Patients exerted maximum effort twice with their left and right hands, with the upper limb lowered parallel to the trunk in the sitting position. The maximum value obtained was used in the analysis. The KEMS was measured using a digital handheld dynamometer (mTasF-1, Anima, Tokyo, Japan). Patients were instructed to remain in the sitting posture, and the dynamometer’s sensor pad was attached to the distal part of the lower leg. The strap from the sensor pad was then attached horizontally to the leg of the platform to fix the hip and knee joints at 90°. Patients were asked to extend the knee joint twice on both sides to push the sensor, and the maximum value was recorded. The KEMS was transformed into newtons adjusted by weight (N/kg). The walking speed was measured using the usual 10 m walk test. Patients were instructed to walk at their usual pace for 14 m, and the middle 10 m was timed. The test was completed twice, and the fastest speed was used in the analysis. The waist circumference was measured in the standing posture, at the height of the navel, and at the time of exhalation using a measuring tape. The functional reach test was measured as the length of the shortened rod after reaching forward as much as possible using the expandable rod [17]. The maximum circumference of the calf was measured with a measuring tape. The maximum calf circumference was measured while sitting without the feet touching the floor.

### 2.6. Cognitive Function and ADL Assessment

Cognitive function was assessed using the Mini-Mental State Examination-Japan (MMSE-J) 3 days before discharge. The MMSE-J comprises 11 categories, with a total score of 30 [18]. Cognitive function was assessed by a therapist in a quiet, private room. The ability of patients to perform ADLs was assessed using the Functional Independence Measure (FIM) [19] at admission and discharge. FIM included 13 motor and 5 cognitive items. Each item had a 7-point ordinal scale from total assistance (a score of 1) to complete independence (a score of 7). Additionally, the FIM gain (the difference between FIM score at discharge and FIM score at admission) and FIM efficiency (FIM gain divided by length of hospitalization) were assessed, which are commonly used to evaluate the degree of improvement.

### 2.7. Rehabilitation Program

The content and duration of the rehabilitation program, including assessment, range of motion exercise, sitting up exercise, sitting exercise, standing exercise, walking exercise, ergometer exercise, resistance training, ADL training, and other parameters recorded every 5 min, were evaluated. A fitness bicycle (Model CMMY-201901; QLEE, Guangzhou, China) was used as the mini-ergometer.

### 2.8. Exercise Training

**Figure 1:**
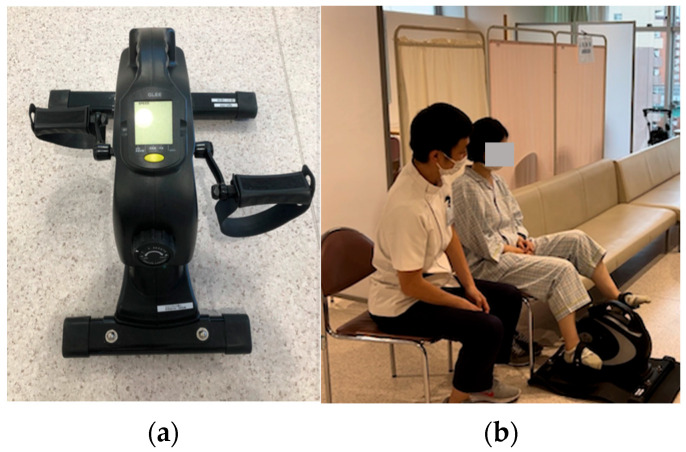
Ergometer exercise. (**a**) The mini-ergometer employed in this study, (**b**) Exercise training with a mini-ergometer.

All patients underwent general cardiac rehabilitation according to the Standard Cardiac Rehabilitation Program for Heart Failure [12]. Specifically, patients underwent rehabilitation in the ward until their general condition stabilized. If they were able to walk steadily, they subsequently received exercise therapy at the rehabilitation center. The study population included a large proportion of elderly individuals, and individualized programs, including training in essential daily living skills, were implemented as follows: Assessment, Range of motion exercise, Sitting up exercise, Sitting exercise, Standing exercise, Walking exercise, Ergometer exercise (Figure 1), Resistance training, and ADL training. Exercise intensity was individually determined, and the rehabilitation sessions were monitored using subjective ratings of perceived exertion based on the Borg scale. All patients underwent cardiac rehabilitation, consisting of 40-to-80 min sessions, five days a week during at admission.

### 2.9. Statistical Analysis

Data were presented as median (interquartile range) for numerical variables and number (percentage) for categorical variables. In between-group comparisons, categorical variables were analyzed using the chi-square test, and numerical variables were analyzed using the Mann–Whitney U test. All analyses were performed using the SPSS 27.0 software package (SPSS Inc., Tokyo, Japan). A *p*-value < 0.05 was considered statistically significant.

## 3. Results

**Figure 2:**
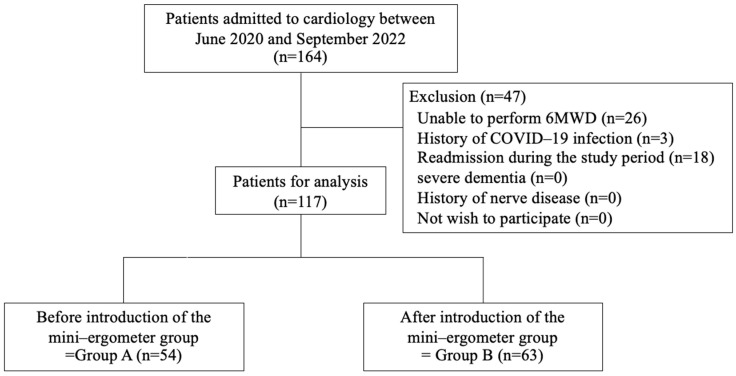
Patient flowchart.

Among 164 patients, 47 were excluded from data analyses. Of the 117 included patients, 54 and 63 were categorized into groups A (range of age, 38–98 years) and B (range of age, 47–98 years), respectively (Figure 2). The demographic data of the patients at admission are presented in Table 1. Group B showed a lower walking speed than Group A at the start of cardiac rehabilitation (Group A: 0.9 m/s vs. Group B: 0.7 m/s, *p* = 0.065). No significant difference in the duration of the cardiac rehabilitation program provided in the ward was observed between the two groups (Group A: 720.0 min vs. Group B: 660.0 min, *p* = 0.109).

Table 2 shows the patients’ clinical characteristics at discharge. Group B showed a significantly higher 6MWD (Group A: 236.0 m vs. Group B: 290.0 m, *p* = 0.020) and a higher walking speed than Group A at discharge (Group A: 0.8 m/s vs. Group B: 0.9 m/s, *p* = 0.084).

Regarding duration of the rehabilitation program during admission, Group B showed a significantly longer ergometer exercise time than Group A (Group A: 0 min vs. Group B: 25.0 min, *p* < 0.001). Group B showed a significantly shorter exercise time for range of motion, sitting, and standing exercises than Group A (range of motion exercise: Group A: 40.0 min vs. Group B: 0 min, *p* < 0.001; sitting exercise: Group A: 0 min vs. Group B: 0 min, *p* = 0.019; standing exercise: Group A: 5.0 min vs. Group B: 0 min, *p* = 0.003) (Table 3).

## 4. Discussion

With restricted access to rehabilitation centers during the COVID-19 pandemic, developing ways to maintain exercise quality and improve physical function during hospitalization was crucial. This study showed a statistically lower tendency in walking speed at the start of cardiac rehabilitation in patients with heart failure after the introduction of the mini-ergometer. However, a rather “higher” walking speed and a higher 6MWD at discharge were observed after the introduction of the mini-ergometer than before the introduction.

The results of this study showed a significantly higher 6MWD after the introduction of the mini-ergometer than before the introduction. The mini-ergometer has (1) the advantage of improving exercise tolerance [4,5], (2) improving walking speed [6], and (3) enhancing muscle strength [7]. The use of a mini-ergometer allowed for the quantification of exercise load, even when access to the rehabilitation center was limited. The introduction of the mini-ergometer might have contributed to the ability to perform sufficient aerobic exercise, which could contribute to the higher 6MWD at discharge. The ability to use a mini-ergometer in a confined space was a factor that allowed sufficient aerobic exercise at the appropriate intensity. In our hospital, aerobic exercise was mainly performed using ergometers before the COVID-19 outbreak. However, aerobic exercise could not be conducted under the restrictions on the rehabilitation center during the early phase of the pandemic. Therefore, the introduction of the mini-ergometer to the ward allowed sufficient time to be dedicated to aerobic exercise. This study showed a tendency toward a higher walking speed after the introduction of the mini-ergometer, which is consistent with previous studies, which reported improvements in walking speed after the use of the mini-ergometer [6]. Taken together, our method might improve physical function at discharge. No significant differences were observed between the groups in terms of age or MMSE-J scores. Previous studies focusing on patients with arterial hypertension have demonstrated that age exerts a significant negative impact on MMSE scores [20]. However, although the patients in both groups of the present study were of advanced age, their MMSE-J scores remained within the normal range.

The statement issued by the Japanese Society of Cardiac Rehabilitation defines it as follows: “Cardiac rehabilitation refers to a long-term, multifaceted, comprehensive program designed to optimize a cardiac patient’s physical, psychological, social, and vocational status, in addition to stabilizing, slowing, or even reversing the progression of the underlying atherosclerotic or heart failure processes, thereby reducing recurrence, rehospitalization and mortality and enabling patients to live comfortably and actively. Cardiac rehabilitation programs include “medical assessment, prescribed exercise training, coronary risk factor modification, patient education, counseling and optimal medical therapy” for individual patients, which are provided by a multidisciplinary team in a coordinated manner [21].” Thus, although cardiac rehabilitation necessitates a multifaceted approach, pharmacological therapy is of particular importance in the acute-phase management of patients with heart failure [22]. It is therefore desirable to provide effective and efficient exercise therapy in conjunction with pharmacological interventions.

Because the provision of cardiac rehabilitation has been restricted due to the COVID-19 pandemic, efforts were needed to ensure that exercise therapy conducted in the ward maintained the same quality as that performed in the rehabilitation center [11,14]. In the rehabilitation program before the introduction of the mini-ergometer, a significant amount of time was spent on a range of motion, sitting, and standing exercises. Designing a rehabilitation program that can serve as an alternative to the ergometer within a confined space of a ward may be challenging. Aerobic exercise without the use of exercise equipment is typically represented by walking [8]. However, in this study, no significant difference in the treatment time of walking exercises for cardiac rehabilitation programs was observed between the two groups. Walking exercises conducted in the ward were sometimes performed as part of the mobilization assessment. Therefore, there might not be enough space in the ward for walking as an aerobic exercise. Since the mini-ergometer does not take up much space, a mini-ergometer was introduced to the ward to provide tailored quantitative and effective exercise prescriptions according to the patients’ condition and physical function. Indeed, its utility in improving physical function in patients with heart failure has been demonstrated.

The social isolation due to the COVID-19 pandemic gradually eased, and life was slowly returning to normal. However, prolonged social isolation might negatively affect the current physical function in older patients with heart failure, as evidenced by the decreased walking speed observed in this study. Patients with heart failure who have a decline in physical function, including walking speed, have been reported to have a poor prognosis in terms of death and readmission [23,24]. The location for cardiac rehabilitation exercise was restricted due to the COVID-19 pandemic. Therefore, taking measures to ensure that the exercise content was not restricted was necessary.

During the COVID-19 pandemic, various lifestyle diseases were affected, such as the deterioration of diabetes control [25]. Additionally, cardiovascular diseases increased [26,27]. Therefore, the role of cardiac rehabilitation is significant. In this study, the rehabilitation methodology is considered highly important and valuable for future infectious disease outbreaks and disasters.

This study has some limitations. First, this was a single-center registry and retrospective study with a small sample size. Second, the different circumstances surrounding the healthcare systems in Japan and other countries might limit the generalizability of the study’s results. Third, the baseline 6MWD was not measured. Therefore, the effect of the mini-ergometer could not be discussed. However, the findings highlighted the importance of implementing measures to maintain the quality of exercise intensity even when access to rehabilitation centers is limited during pandemics.

## 5. Conclusions

The findings of this study demonstrate that the introduction of a mini-ergometer to the ward could ensure quantitative exercise intensity even under restrictions on access to rehabilitation centers during the COVID-19 outbreak, leading to improvements in physical function (6MWD, Walking speed) at discharge in patients with heart failure. The rehabilitation methodology is regarded as highly important and valuable for addressing future infectious disease outbreaks and disasters.

## Figures and Tables

**Table 1:** Patients’ demographic and clinical characteristics at admission before and after the introduction of the mini-ergometer.

	Before the Introduction of the Mini-Ergometer (Group A) (*n* = 54)	After the Introduction of the Mini-Ergometer (Group B) (*n* = 63)	*p*
Age, years	83.0 (73.0–88.3)	84.0 (73.0–87.0)	0.540
Gender (male/female), *n*	28/26	34/29	0.819
Body mass index, kg/m^2^	21.3 (18.1–23.6)	22.3 (19.7–24.7)	0.124
NYHA functional class			
Class I	7	8	0.966
Class II	26	22	0.147
Class III	16	27	0.139
Class IV	5	6	0.961
Comorbidities			
Hypertension	32	44	0.232
Dyslipidemia	14	22	0.293
Diabetes Mellitus	17	32	0.035
Myocardial Infarction	9	16	0.251
Cerebral Infarction	9	14	0.451
Cerebral Hemorrhage	2	3	0.778
Cancer	9	14	0.451
Chronic Kidney Disease	14	11	0.265
Medication			
Renin-angiotensin system inhibitor	12	3	0.863
Beta blocker	35	46	0.338
Calcium channel blocker	12	16	0.688
Loop diuretic	46	46	0.109
Thiazide diuretic	0	1	0.353
V2 receptor antagonist	21	32	0.197
Mineralocorticoid Receptor Antagonist	15	26	0.127
sodium-glucose cotransporter 2 inhibitor	8	15	0.222
Days before the initiation of the rehabilitation program, days	2.5 (2.0–4.3)	3.0 (2.0–4.0)	0.188
Length of hospitalization, days	19.0 (14.0–29.5)	18.0 (14.0–24.0)	0.749
Rehabilitation place, min	1310/185 (87.6/12.4)	1351/196 (87.3/12.5)	0.806
Ward	720.0 (420.0–1425.0)	660.0 (390.0–840.0)	0.109
Rehabilitation center	40.0 (0–120.0)	60.0 (0–180.0)	0.163
SBP, mmHg	139.0 (120.0–161.0)	136.0 (120.0–160.0)	0.716
NT-proBNP, pg/dL	5103 (2847–10,097)	3392 (1773–7034)	0.052
GNRI	94.5 (84.1–101.0)	95.5 (83.5–102.9)	0.707
Serum hemoglobin, g/dL	11.7 (10.2–13.5)	12.2 (10.6–13.6)	0.202
Serum albumin, g/dL	3.6 (3.2–3.9)	3.6 (3.3–3.9)	0.695
Serum sodium, mEq/L	141.0 (137.5–144.0)	140.0 (138.0–143.0)	0.541
eGFR, ml/min/1.73 m^2^	47.2 (38.6–61.1)	50.0 (36.1–62.6)	0.688
LVEF, %	41.7 (29.9–60.1)	38.3 (27.9–55.8)	0.330
Grip strength, kg	18.3 (13.9–23.0)	19.3 (14.9–25.9)	0.367
Walking speed, m/s *	0.9 (0.7–1.0)	0.7 (0.5–0.9)	0.065
FIM score, points			
Total	97.5 (78.0–114.0)	105.0 (86.0–120.0)	0.153
Motor	69.0 (53.3–79.0)	71.0 (57.0–85.0)	0.141
Cognitive	35.0 (25.0–35.0)	35.0 (28.0–35.0)	0.190

FIM, Functional Independence Measure; GNRI, geriatric nutritional risk index; eGFR, estimated glomerular filtration rate; LVEF, left ventricular ejection fraction; NT-proBNP, N-terminal pro-brain natriuretic peptide; NYHA, New York Heart Association; SBP, systolic blood pressure. * The walking speed was calculated using the usual 10 m walk test.

**Table 2:** Patients’ clinical characteristics at discharge before and after the introduction of the mini-ergometer.

	Before the Introduction of the Mini-Ergometer (Group A) (*n* = 54)	After the Introduction of the Mini-Ergometer (Group B) (*n* = 63)	*p*
Grip strength, kg	18.5 (13.3–24.8)	21.0 (14.9–27.1)	0.163
Walking speed, m/s	0.8 (0.6–1.0)	0.9 (0.7–1.1)	0.084
Waist circumference, cm	81.0 (72.0–88.5)	85.1 (74.5–91.9)	0.178
KEMS, N/kg	3.3 (2.6–4.2)	3.6 (2.5–4.3)	0.779
6MWD, m	236.0 (163.8–312.5)	290.0 (214.5–350.0)	0.020
FRT, cm	26.0 (19.8–33.5)	30.0 (23.0–33.3)	0.144
MMSE-J	27.0 (22.0–28.5)	26.0 (23.0–28.0)	0.758
FIM score			
Total	114.5 (95.8–125.3)	120.0 (108.0–125.0)	0.154
Motor	80.5 (70.0–90.3)	85.0 (76.0–90.0)	0.153
Cognitive	35.0 (25.0–35.0)	35.0 (31.0–35.0)	0.105
FIM gain	11.5 (0–23.0)	8.0 (0–25.0)	0.949
FIM efficiency	0.5 (0–0.9)	0.5 (0–0.8)	0.920

Data were presented as median (interquartile range). FIM, Functional Independence Measure; FRT, functional reach test; KEMS, knee extensor muscle strength; MMSE, Mini-Mental State Examination; 6MWD, 6 min walking distance.

**Table 3:** Duration (min) of the rehabilitation program during admission. Data were presented as median (interquartile range). ADL, activities of daily living.

	Before the Introduction of the Mini-Ergometer (Group A) (*n* = 54)	After the Introduction of the Mini-Ergometer (Group B) (*n* = 63)	*p*
Assessment	250.0 (195.0–340.0)	260.0 (193.8–306.3)	0.600
Range of motion exercise	40.0 (8.8–107.5)	0 (0–25.0)	<0.001
Sitting up exercise	0 (0–0)	0 (0–0)	0.556
Sitting exercise	0 (0–35.0)	0 (0–0)	0.019
Standing exercise	5.0 (0–50.0)	0 (0–5.0)	0.003
Walking exercise	180.0 (81.3–402.5)	180.0 (100.0–280.0)	0.382
Ergometer exercise	0 (0–2.5)	25.0 (0–70.0)	<0.001
Resistance training	165.0 (78.8–327.5)	150.0 (95.0–240.0)	0.392
ADL training	0 (0–55.0)	0 (0–20.0)	0.313
Others	27.5 (5.0–67.5)	10.0 (0–30.0)	0.035
Total	790.0 (535.0–1550.0)	720.0 (480.0–940.0)	0.141

## Data Availability

The data that support the findings of this study are available from the corresponding author upon reasonable request.

## Abbreviations

The following abbreviations are used in this manuscript:

6MWD	6 min walking distance
ADL	activities of daily living
KEMS	knee extensor muscle strength
FIM	Functional Independence Measure
GNRI	geriatric nutritional risk index
eGFR	estimated glomerular filtration rate
LVEF	left ventricular ejection fraction
NT-pro BNP	N-terminal pro-brain natriuretic peptide
NYHA	New York Heart Association
SBP	systolic blood pressure
FRT	functional reach test
MMSE	Mini-Mental State Examination
